# Metabolic Changes Precede the Development of Pulmonary Hypertension in the Monocrotaline Exposed Rat Lung

**DOI:** 10.1371/journal.pone.0150480

**Published:** 2016-03-03

**Authors:** Olga Rafikova, Mary L. Meadows, Jason M. Kinchen, Robert P. Mohney, Emin Maltepe, Ankit A. Desai, Jason X.-J. Yuan, Joe G. N. Garcia, Jeffrey R. Fineman, Ruslan Rafikov, Stephen M. Black

**Affiliations:** 1 Division of Translational and Regenerative Medicine, The University of Arizona, Tucson, Arizona, United States of America; 2 Department of Medicine, The University of Arizona, Tucson, Arizona, United States of America; 3 Vascular Biology Center, Georgia Regents University, Augusta, Georgia, United States of America; 4 Metabolon, Durham, North Carolina, United States of America; 5 Division of Neonatology, University of California San Francisco, San Francisco, California, United States of America; 6 Department of Pediatrics, University of California San Francisco, San Francisco, California, United States of America; 7 Cardiovascular Research Institute, University of California San Francisco, San Francisco, California, United States of America; Cincinnati Children's Hospital Medical Center, UNITED STATES

## Abstract

There is increasing interest in the potential for metabolic profiling to evaluate the progression of pulmonary hypertension (PH). However, a detailed analysis of the metabolic changes in lungs at the early stage of PH, characterized by increased pulmonary artery pressure but prior to the development of right ventricle hypertrophy and failure, is lacking in a preclinical animal model of PH. Thus, we undertook a study using rats 14 days after exposure to monocrotaline (MCT), to determine whether we could identify early stage metabolic changes prior to the manifestation of developed PH. We observed changes in multiple pathways associated with the development of PH, including activated glycolysis, increased markers of proliferation, disruptions in carnitine homeostasis, increased inflammatory and fibrosis biomarkers, and a reduction in glutathione biosynthesis. Further, our global metabolic profile data compare favorably with prior work carried out in humans with PH. We conclude that despite the MCT-model not recapitulating all the structural changes associated with humans with advanced PH, including endothelial cell proliferation and the formation of plexiform lesions, it is very similar at a metabolic level. Thus, we suggest that despite its limitations it can still serve as a useful preclinical model for the study of PH.

## Introduction

Pulmonary hypertension (PH) is a disease characterized by increased proliferation of the vascular wall leading to increased pulmonary artery pressure that results in right ventricle hypertrophy and subsequent heart failure. However, these symptoms become pronounced only at the late stage of the disease when available treatments already have a modest effect on disease progression. Therefore, the discovery of early markers that predict the development of pulmonary hypertension has important clinical utility. While several pre-clinical models are available to study PH in rodents (induced by chronic hypoxia [1], chronic hypoxia in combination with the VEGFR2 inhibitor SU5416 [2] and the injection of monocrotaline [3]) and in lambs and calves (models of increased pulmonary flow), the underlying concern with all animal models is how well they recapitulate the effects of human disease. One of the most published models is the induction of PH in rats is the monocrotaline model, where a single administration of the plant toxin crotaline induces increased pulmonary pressure and right ventricle hypertrophy within 4-weeks. However, several differences in disease progression (compared to observations in PH patients) has raised to concerns regarding the utility of this model, including the proliferation of mainly smooth muscle cells (without significant endothelial cell proliferation) and the development of concentric lesions in the lung (without the characteristic plexiform lesions seen in the later stages of human PH [4, 5]). However, we postulated that since early changes in the monocrotaline model are similar to those that occur during the initial steps of human disease, this model would be amenable to a metabolic profiling analysis to search for potential biomarkers of early stage PH.

Recent work has utilized metabolomic profiling of PH patients to try and identify useful biomarkers [6, 7]. In this study, we undertook a metabolomic profiling study to determine whether it is possible to identify biomarkers that are present prior to the development of PH, but that have known linkages to pathways that are deranged as the pulmonary hypertensive phenotype progresses. In addition, we wished to see how the metabolomics profile of the rat MCT model of PH compared to previously reported metabolic data for PH patients. Our data indicate that 14 days after MCT injection, and before obvious PH has developed, we could clearly identify significant changes in glycolysis, carnitine homeostasis, alterations in biomarkers related to cell proliferation, inflammation and fibrosis, and reductions in glutathione synthesis, all of which are known to be associated with the progression of PH [8–13]. Further, we identified significant similarities between our data and published data on the global metabolic profile obtained from patient’s lungs with PH, suggesting that despite its failure to recapitulate all the structural characteristics of human PH, the MCT model recapitulates much of the metabolic changes occurring during the development of PH.

## Methods

### Metabolic studies

A total of 20 male Sprague Dawley rats (SD; 220-270g) were used in this study (n = 10 per group). Control group received vehicle for monocrotaline (MCT). Pre-pulmonary hypertension (PH) group received a single injection of MCT (60 mg/kg i.p.) to induce and were sacrificed after 14 days. For this purpose rats were anesthetized (Inactin, 100 mg/kg i.p.), a PE-240 polyethylene tube was inserted into the trachea and connected to a Harvard Rodent Ventilator (Model 683; Harvard Apparatus, South Natick, MA) to facilitate breathing. The thorax was opened, the cut in ascending aorta was made and the lungs were flashed with saline (0.9% sodium chloride) via the needle inserted into right ventricle to remove the blood from pulmonary vessels. Animals were euthanized by an anesthetic overdose, lungs were removed and snap frozen in liquid nitrogen then stored at -80°C until being sent to Metabolon for analysis.

### Acute measurement of hemodynamic parameters

An additional set of animals (n = 8 per group) consisting of control rats, rats injected with MCT and sacrificed after 14 days (pre-PH group), and rats injected with MCT and sacrificed after 28 days (PH group) were used to measure right ventricle (RV) hemodynamics and RV hypertrophy. Briefly, a PE-240 polyethylene tube was inserted into the trachea to facilitate breathing. A customized pressure transducer catheter (SPR-513, Millar Instruments, Houston, TX), connected to a Millar Transducer Control Unit TC-510 and PL3504 PowerLab 4/35 data acquisition system (ADInstruments, Inc., Colorado Springs, CO) was inserted into the RV via the right jugular vein and right atrium. A 30min stabilization period was permitted before a 30min recording of the right ventricular pressure was conducted. At the end of pressure recording, the animals were euthanized by an anesthetic overdose, and the heart and lungs were dissected and weighed. The right ventricle free wall (RV) was separated from the left ventricle and septum (LV+S) to determine the wet weights and the RV to LV+S weight ratio (RV/LV+S).

### Ethics Statement

Animals were housed in the Georgia Regents University animal care facility for at least 1 week before being used in the experiments. Animals were kept in a 12-hour light/dark cycle at an ambient temperature of 22°C and received standard rodent food and water ad libitum. Animals were housed in the Georgia Regents University animal care facility for at least 1 week before being used in the experiments. Animals were kept in a 12-hour light/dark cycle at an ambient temperature of 22°C and received standard rodent food and water ad libitum. All experimental procedures were approved by the Institutional Animal Care and Use Committee at Georgia Regents University. Animals were euthanized by an anesthetic overdose.

### Sample Accessioning

Each lung sample was accessioned into the Metabolon laboratory management information system (LIMS) system and assigned a unique identifier associated with the original source identifier only. This identifier was used to track all sample handling, tasks, results, etc. The samples were tracked by the LIMS system. All portions of any sample were automatically assigned their own unique identifiers by LIMS when a new task was created; the relationship of these samples was also tracked. All samples were maintained at -80C until processed.

### Sample Preparation

Samples were prepared using the automated MicroLab STAR^®^ system from the Hamilton Company. A recovery standard was added prior to the first step in the extraction process for QC purposes. To remove protein, dissociate small molecules bound to protein or trapped in the precipitated protein matrix, and to recover chemically diverse metabolites, proteins were precipitated with methanol under vigorous shaking for 2 min (Glen Mills GenoGrinder 2000) followed by centrifugation. The resulting extract was divided into five fractions: one for analysis by UPLC-MS/MS with positive ion mode electrospray ionization, one for analysis by UPLC-MS/MS with negative ion mode electrospray ionization, one for analysis by UPLC-MS/MS polar platform (negative ionization), one for analysis by GC-MS, and one sample was reserved for backup. Samples were placed briefly on a TurboVap^®^ (Zymark) to remove the organic solvent. For LC, the samples were stored overnight under nitrogen before preparation for analysis. For GC, each sample was dried under vacuum overnight before preparation for analysis.

### QA/QC

Several types of controls were analyzed in concert with the experimental samples: a pooled matrix sample generated by taking a small volume of each experimental sample served as a technical replicate throughout the data set; extracted water samples served as process blanks; and a cocktail of QC standards carefully chosen not to interfere with the measurement of endogenous compounds were spiked into every analyzed sample. This allows instrument performance monitoring and aided chromatographic alignment. Instrument variability was determined by calculating the median relative standard deviation (RSD) for the standards added to each sample prior to injection into the mass spectrometers. Overall process variability was determined by calculating the median RSD for all endogenous metabolites (i.e., non-instrument standards) present in 100% of the pooled matrix samples. Experimental samples were randomized across the platform run with QC samples spaced evenly among the injections.

### Ultrahigh Performance Liquid Chromatography-Tandem Mass Spectroscopy (UPLC-MS/MS)

The LC/MS portion of the platform was based on a Waters ACQUITY ultra-performance liquid chromatography (UPLC) and a Thermo Scientific Q-Exactive high resolution/accurate mass spectrometer interfaced with a heated electrospray ionization (HESI-II) source and Orbitrap mass analyzer operated at 35,000 mass resolution. The sample extract was dried then reconstituted in acidic or basic LC-compatible solvents, each of which contained 8 or more injection standards at fixed concentrations to ensure injection and chromatographic consistency. One aliquot was analyzed using acidic positive ion-optimized conditions and the other using basic negative ion-optimized conditions in two independent injections using separate dedicated columns (Waters UPLC BEH C18-2.1x100 mm, 1.7 μm). Extracts reconstituted in acidic conditions, were gradient eluted from a C18 column using water and methanol containing 0.1% formic acid. Basic extracts were similarly eluted from a C18 column using methanol and water, in the presence of 6.5mM ammonium bicarbonate. The third aliquot was analyzed via negative ionization following elution from a HILIC column (Waters UPLC BEH Amide 2.1x150 mm, 1.7 μm) using a gradient consisting of water and acetonitrile with 10mM ammonium formate. The MS analysis alternated between MS and data-dependent MS/MS scans using dynamic exclusion, and the scan range was from 80–1000 m/z.

### Gas Chromatography-Mass Spectroscopy (GC-MS)

The samples for GC-MS analysis were dried under vacuum for a minimum of 18h prior to being derivatized under dried nitrogen using bistrimethyl-silyltrifluoroacetamide. Derivatized samples were separated on a 5% diphenyl/95% dimethyl polysiloxane fused silica column (20m x 0.18mm ID; 0.18μm film thickness) with helium as a carrier gas and using a temperature ramp from 60°C to 340°C in a 17.5 min period. Samples were analyzed using a Thermo-Finnigan Trace DSQ fast-scanning single-quadrupole mass spectrometer with an electron impact ionization (EI) and operated at unit mass resolving power. The scan range was 50–750 m/z.

### Bioinformatics

The informatics system used consisted of four major components, the LIMS, the data extraction and peak-identification software, data processing tools for QC and compound identification, and a collection of information interpretation and visualization tools for use by data analysts. The hardware and software foundations for these informatics components were the LAN backbone, and a database server running Oracle 10.2.0.1 Enterprise Edition.

### Data Extraction and Compound Identification

Raw data was extracted, peak-identified and QC processed using Metabolon’s hardware and software. Compounds were identified by comparison to library entries of purified standards. Biochemical identifications are based on three criteria: retention index within a narrow RI window of the proposed identification, accurate mass match to the library +/- 0.005 amu, and the MS/MS forward and reverse scores between the experimental data and authentic standards. The MS/MS scores are based on a comparison of the ions present in the experimental spectrum to the ions present in the library spectrum.

### Statistical Calculations

Two types of statistical analysis were performed: (1) significance tests and (2) classification analysis. Standard statistical analyses were performed in ArrayStudio (OmicSoft Corporation) on log transformed data. For those analyses not standard in ArrayStudio, the programs R (http://cran.r-project.org/) or JMP (SAS Institute Inc.) were used. Welch’s two-sample t-test was used to test whether two unknown means are different from two independent populations. The False Discovery Rate (FDR) was also determined to account for the possibility of false positives present in large data sets. The FDR for a given set of compounds was estimated using the q-value [14]. The Random forest supervised classification technique based on an ensemble of decision trees [15] was also used to generating the ““out-of-bag” (OOB) error rate” as a measure of prediction accuracy. P and q value is provided for each metabolite in S1 Table.

## Results

### Validation of the early stage of PH

**Fig 1:**
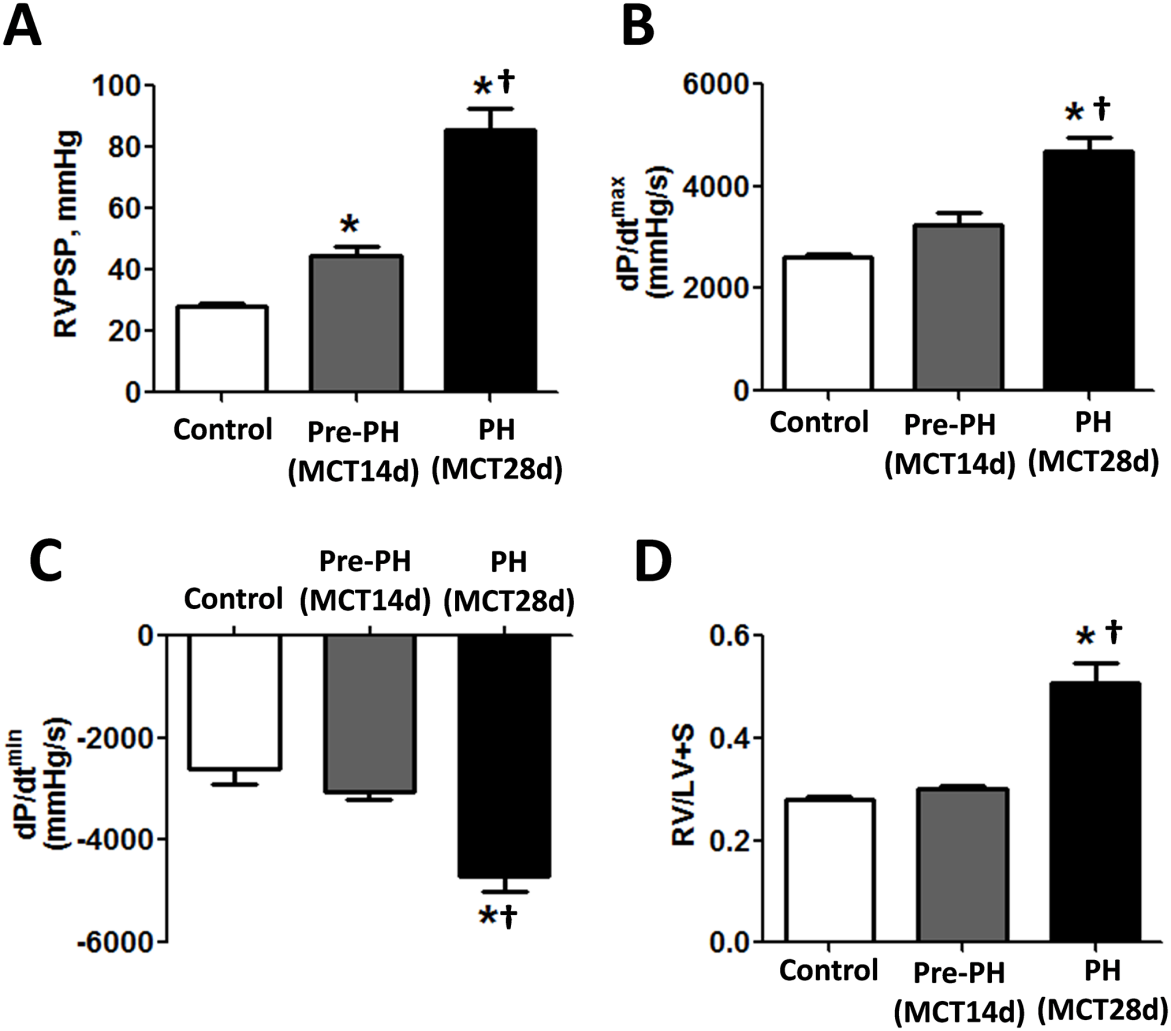
Right ventricle hemodynamic changes and right ventricle hypertrophy at early and developed stages of pulmonary hypertension induced by MCT injection in the rat. The developed stage of PH (28 days after MCT injection) was characterized by a significant increase in RVPSP (A), increased RV load, assessed by RV contractility (B) and RV relaxation (C) and severe RV hypertrophy. However, the early stage of PH (pre-PH) 14 days after MCT injection exhibits only a mild increase in RVPSP with no alterations in RV function and hypertrophy (A-C). Results are expressed as mean ± SEM; n = 6–8. *P<0.05 vs. Control group; ^†^P<0.05 vs. Pre-PH (MCT14days) group.

Our hemodynamic data, collected after 14 days of MCT treatment, demonstrate a significant increase in right ventricle peak systolic pressure (RVPSP), and indicate that at this early time point there is already active pulmonary vasoconstriction (Fig 1A). However, this increase in pressure was found to be relatively mild when compared to 28 days of MCT exposure, which in normally used to induce an established stage of PH (Fig 1A). Moreover, the increase in pulmonary pressure, although significant, did not induce significant changes in right ventricle workload, since it did not significantly alter either right ventricle contractility or relaxation (Fig 1B and 1C). The Fulton index (RV/LV+S) also did not identify any signs of RV hypertrophy 14 days after MCT, while at 28 days there was a significant evidence of RV hypertrophy (Fig 1D). Thus, 14 days of MCT exposure induces a very early stage of PH (pre-PH) that exhibits only pulmonary vasoconstriction with no significant changes in the primary determinants of developed PH, RV work load and hypertrophy.

### Overall statistics of metabolic study

**Fig 2:**
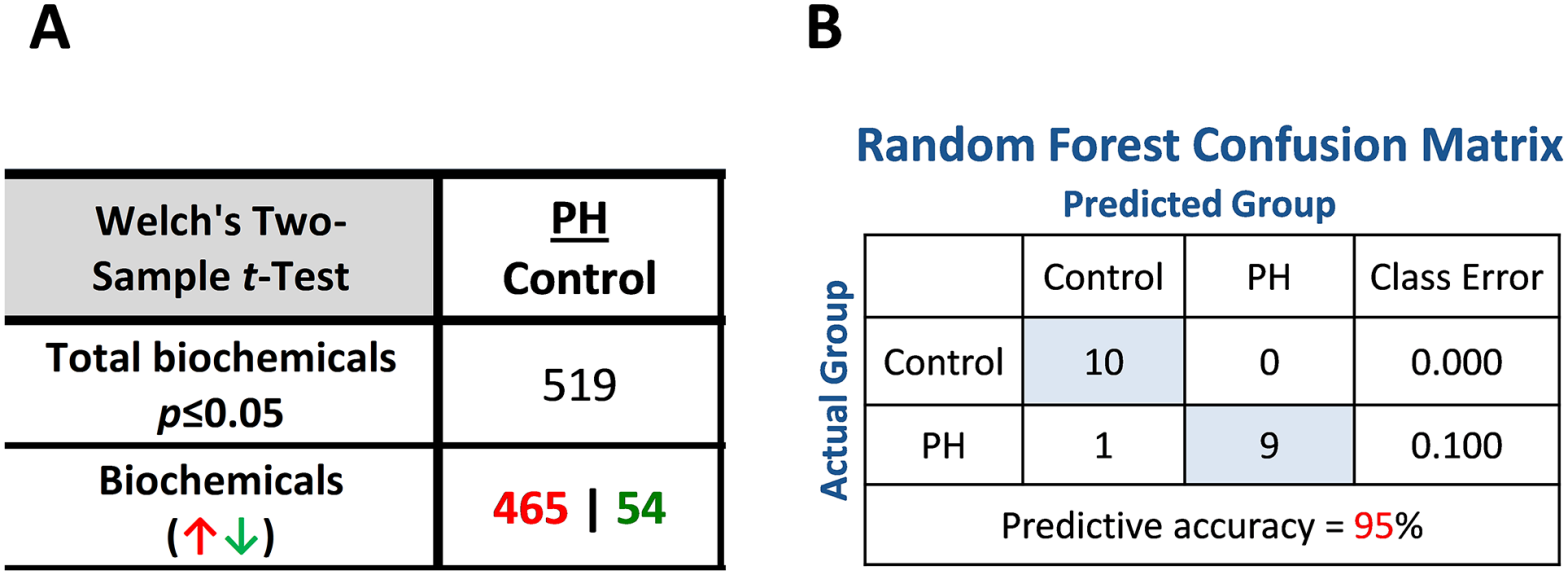
Overall metabolic profiling statistics. Statistical comparisons using Welch’s Two-Sample t-Test show significantly altered metabolic changes in MCT-treated lungs (N = 10) compared with controls (N = 10, A). MCT significantly increased a large number of metabolites (465) but only significantly decreased 54 metabolites compared to the normal lung (p<0.05). Random forest analysis for control vs. MCT-treated rats yielded a predictive accuracy of 95% (B).

In this study, the metabolic profiles of lung samples from either Control (n = 10) or PH (n = 10) MCT-treated rats (14 days after MCT single injection) were compared. The presented dataset was comprised of a total of 789 compounds of known identity. Our data indicate that 519 biomarkers were altered with a significant p-value where 465 biomarkers were increased and 54 were decreased compared to control (Fig 2A). Random forest analysis for control vs. pre-PH yielded a predictive accuracy of 95%; one PH sample was incorrectly sorted, perhaps due to variation within the disease model (Fig 2B). An estimate of the false discovery rate (q-value) was calculated to take into account the multiple comparisons that normally occur in metabolomic-based studies (S1 Table). To exclude compounds that are meeting the p<0.05 cut-off by random chance, the q-value are shown. A low q-value (q<0.10) is an indication of high confidence in a result. In our data using a cut-off at q<0.1 indicates no false positive results.

### Altered bioenergetics

**Fig 3:**
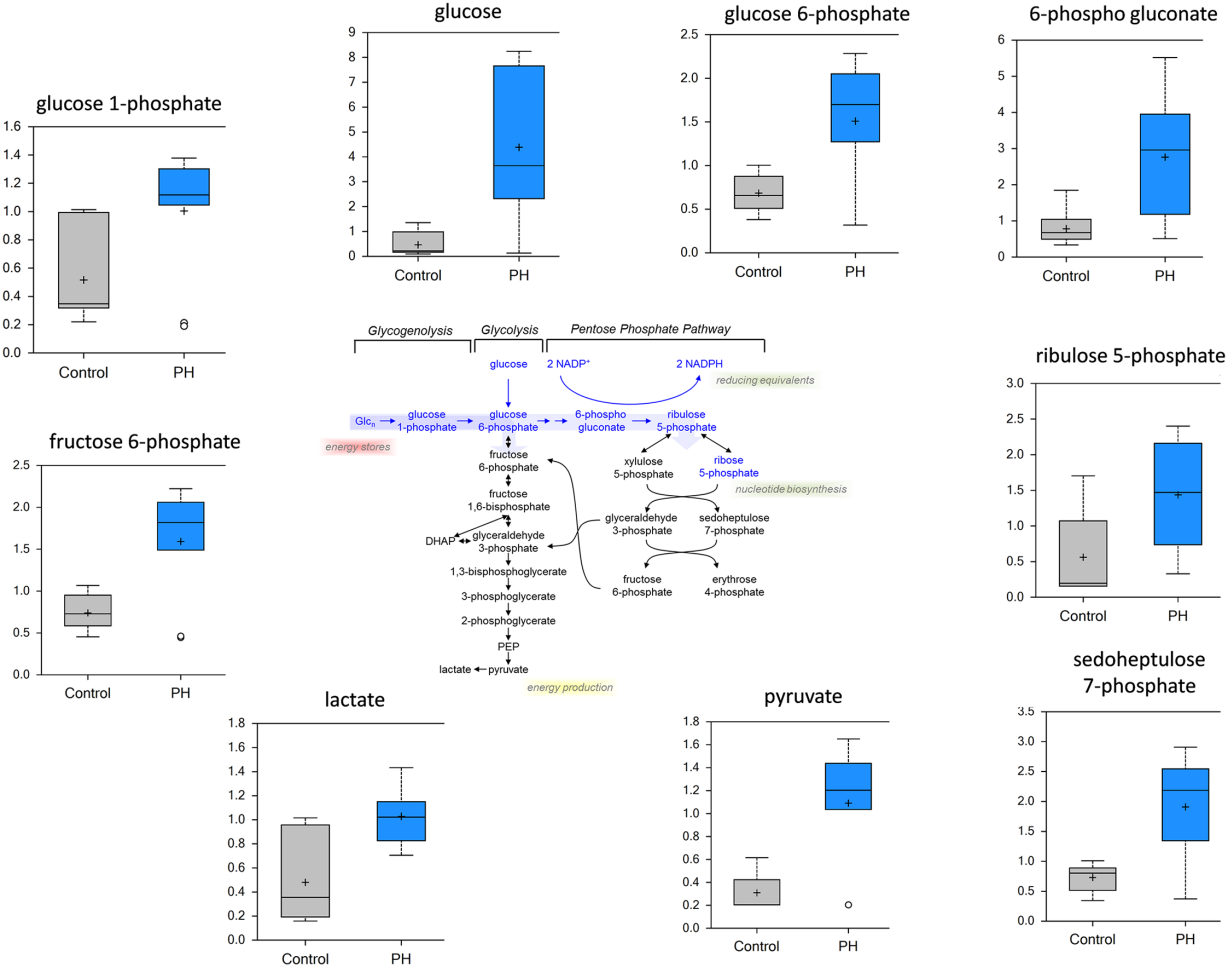
Evidence for a glycolytic switch in the MCT-treated rat lung. Data for control lung are represented in grey boxes and data for the pre-PH lung are represented in blue boxes. Quantities are in arbitrary units (N = 10, p<0.05). MCT-treated animals have higher levels of glucose, glucose-6-phosphate, glucose-1-phosphate, fructose, fructose-6-phosphate, 6-phospho gluconate, ribulose 5-phosphate, sedoheptulose-7-phosphate, pyruvate and lactate. These metabolic data are consistent with an upregulation of glycolytic or glucose dependent pathways in the lungs of pre-PH rats.

Several studies have suggested that a Warburg-like switch from oxidative phosphorylation to glycolysis is characteristic of PH [16, 17]. Thus, we initially examined if we could identify changes in glycolysis in the MCT rats prior to the development of PH. Our data indicate that, compared to control group, there are significant elevations in glucose (~10-fold), glycolytic intermediates (glucose 6-phosphate and fructose 6-phosphate) and glycolytic products (pyruvate and lactate) (Fig 3). We also found that pentose phosphate pathway metabolites (6-phosphogluconate and sedoheptulose-7-phosphate) were also increased (Fig 3). Metabolites of glycogen breakdown—glucose 1-phosphate (Fig 3), maltotetraose, maltotriose and maltose (data not shown)–were also increased in pre-PH lung. Elevated levels of Pentose Phosphate pathway metabolites (ribose 5 phosphate and sedoheptulose 7 phosphate) are suggestive of increased glucose uptake and utilization in PH. This also contribute to increased nucleic acid synthesis) along with erythritol and xylitol (aromatic amino acid synthesis). The ribulose/xylulose-5 phosphate ratio suggests that nucleotide synthesis is preferred, which would also generate NADPH as a reducing agent. Thus, increased flux through the PPP may be in response to increased oxidative stress in PH. Taken together, these data are consistent with a metabolic shift toward glycolysis and suggest increased glucose uptake and utilization is an early marker that precedes the development of PH.

### Disrupted carnitine homeostasis

**Fig 4:**
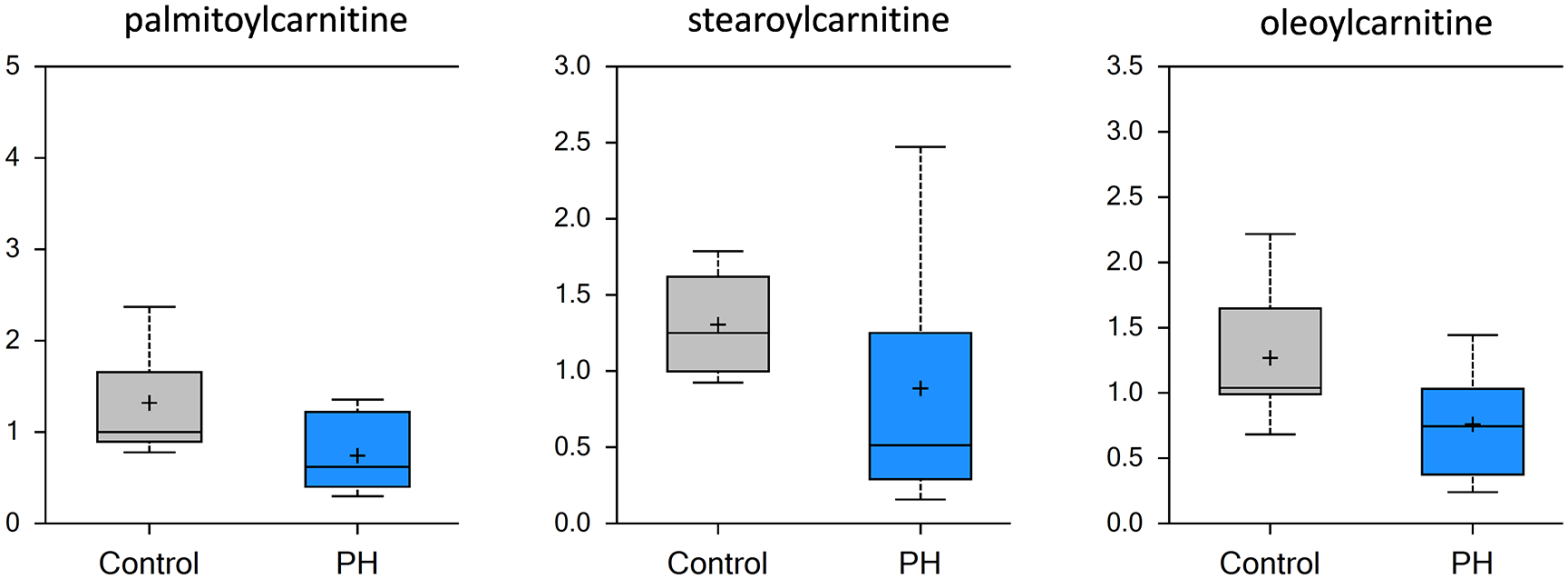
Carnitine homeostasis is altered in the MCT-treated rat lung. Significant decreases in conjugated acyl carnitines such as palmitoylcarnitine, stearoylcarnitine and oleoylcarnitine indicate that there is disrupted fatty acid transport to mitochondria in the lungs of pre-PH rats (N = 10, p<0.5).

To undergo beta-oxidation in the mitochondria, long-chain fatty acids must first be conjugated to carnitine to form long-chain acylcarnitines. Prior work has linked derangements in carnitine homeostasis with the development of pulmonary hypertension [10, 18, 19]. While long chain fatty acids were not significantly changed as a class, we identified significantly decreased levels of long-chain acylcarnitines (palmitoylcarnitine, stearoylcarnitine, linoleoylcarnitine, and oleoylcarnitine) in the pre-PH lung (Fig 4). This suggests that there is a reduced utilization of fatty acids for beta-oxidation, though the ketone body 3-hydroxybutyrate (BHBA) was significantly increased (3.02 fold, data not shown) pointing again on increased glucose metabolism that can produce BHBA. Taken together, these data are again indicative that fatty acid beta-oxidation is altered prior to the development of PH and, in conjunction with increased glycolytic products, are consistent with a Warburg-like metabolic shift.

### Increased inflammatory biomarkers

**Fig 5:**
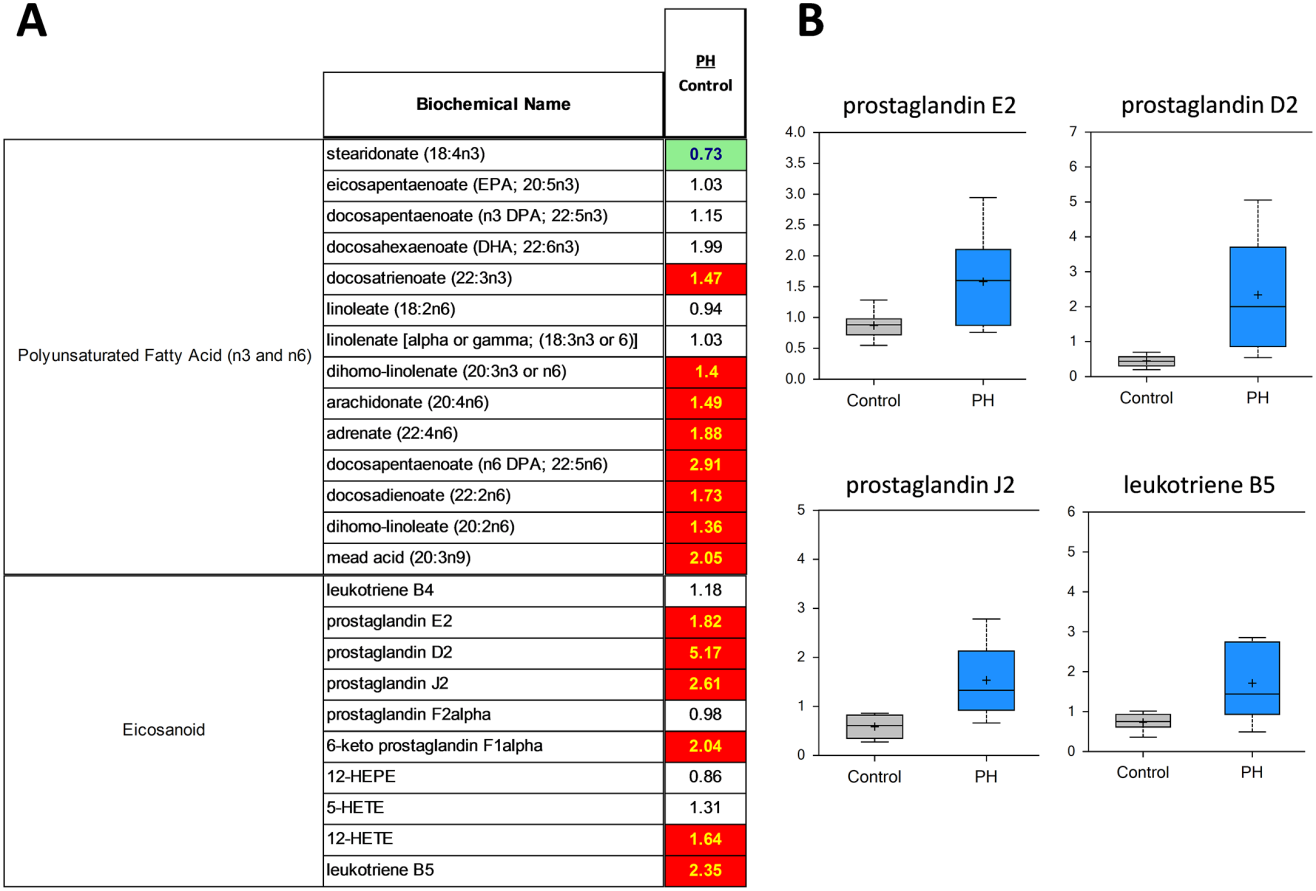
A shift in the balance omega 6 and omega 3 fatty acids and increased pro-inflammatory eicosanoid production in the MCT-treated rat lung. The ratio between pre-PH and control metabolites show both significant increases (red boxes) for omega 6, omega 3 fatty acids and eicosanoids and a trending decrease (light green box) for stearidonate (A). The pro-inflammatory prostaglandins E2, D2, J2 and leukotriene B5 are also significantly increased in the pre-PH lung (N = 10, p<0.05, B).

**Fig 6:**
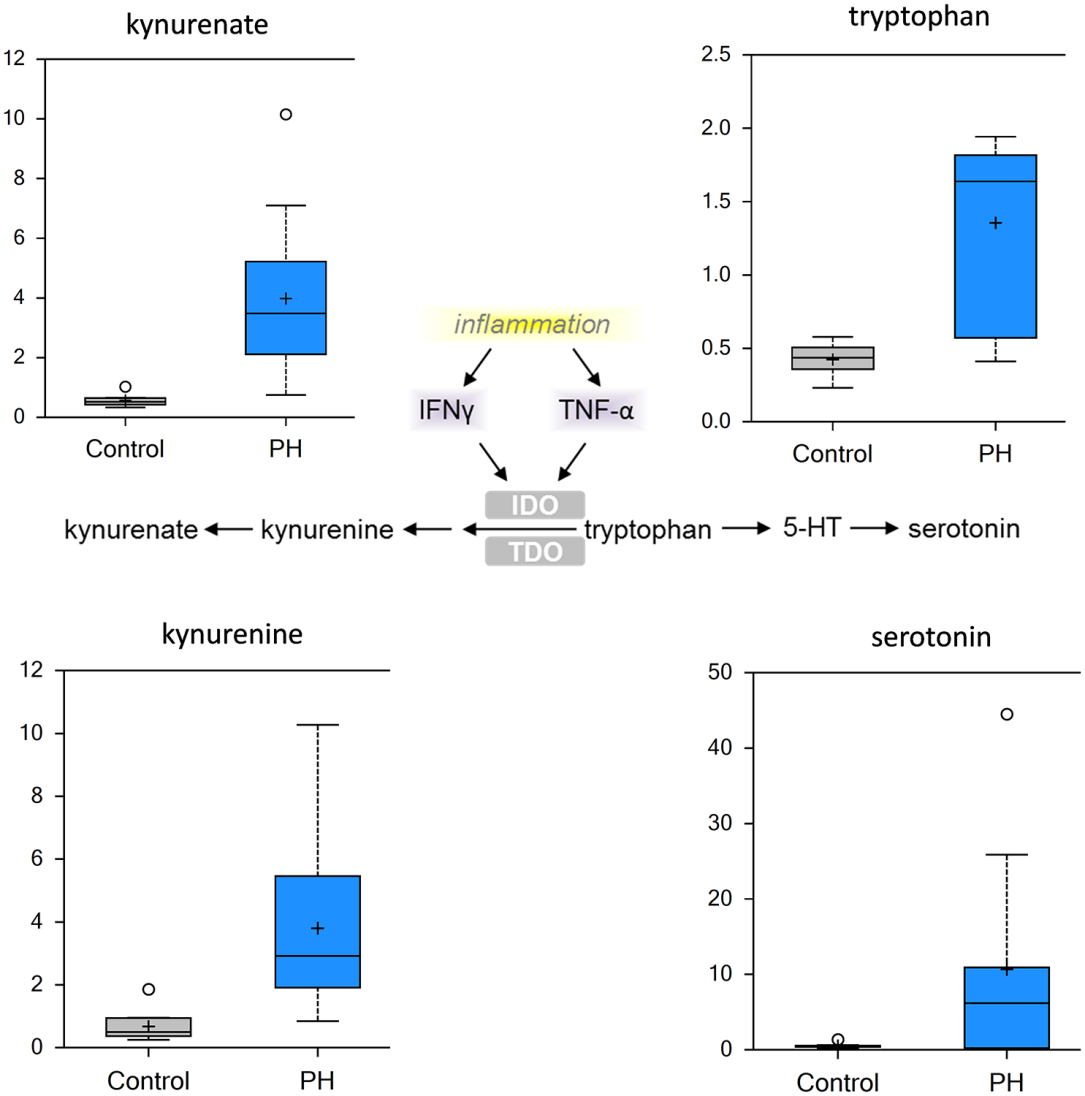
Inflammatory metabolites are increased in the MCT-treated rat lung. Inflammation, via INF-gamma or TNF-alpha, activates indoleamine-2,3-dioxygenase (IDO) or tryptophan-2,3-dioxygenase (TDO) that degrade tryptophan to kynurenine/kynurenate (center pathway). There is a significant increase in tryptophan, kynurenine, kynurenate and serotonin in the pre-PH lung suggestive of increased inflammation. (N = 10, p<0.05).

**Fig 7:**
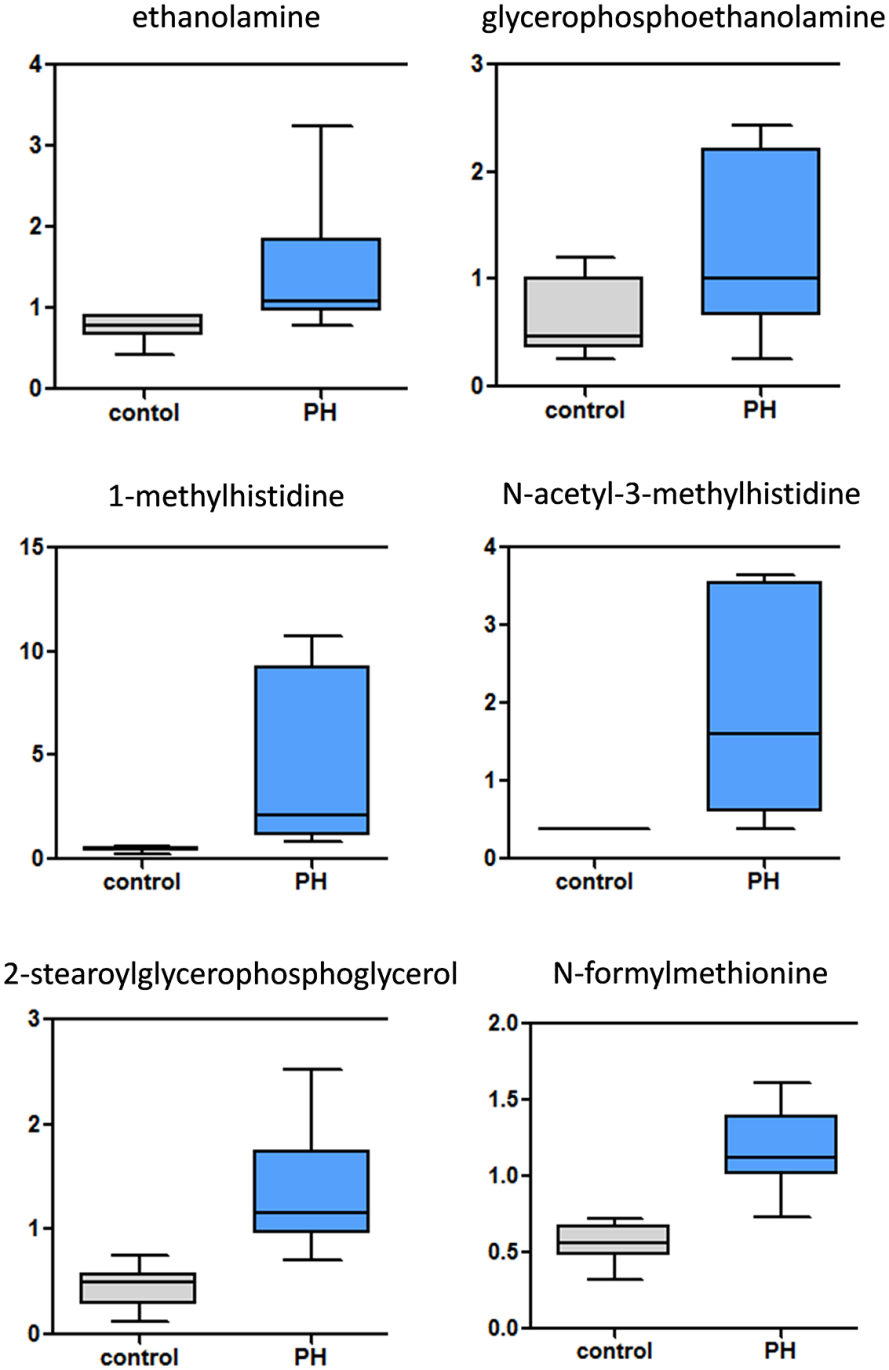
Biomarkers of cellular damage are increased in the MCT-treated rat lung. Phospholipid degradation and membrane remodeling markers (ethanolamine and glycerophosphoethanolamine) are significantly increased in the pre-PH lung. Methylhistidines (1-methylhhistidine and N-acetyl-3-histidine), produced by methylation of actin and myosin in muscle, are indicative of muscle protein breakdown and therefore muscular damage. Significant increases in mitochondrial membrane degradation (2-stearoylglycerophosphoglycerol) and the breakdown product of mitochondrially-encoded/synthesized proteins (N-formylmethionine) reflect mitochondrial damage. (N = 10, p<0.05).

As might be expected from the etiology of pulmonary hypertension, markers of inflammation, stress, tissue remodeling and redox homeostasis were substantially altered in PH. Omega-6 fatty acids (for example, arachidonate, docosadienoate and dihomo-linoleate), which are precursor compounds for prostaglandin biosynthesis, were increased in the pre-PH lung (Fig 5A). These metabolites can be further processed by lipoxygenase (LOX) and cyclooxygenase (COX) enzymes to generate inflammatory eicosanoids, such as prostaglandin E2, prostaglandin D2, prostaglandin J2, and leukotriene B5, all of which were increased in pre-PH lungs (Fig 5B). Further, the accumulation of kynurenine/kynurenate (Fig 6) is again indicative that a highly inflammatory state exists prior to the development of PH. The significant increase in serotonin (Fig 7) and histamine (S1 Table) are also interesting as they have been associated with the development of PH [20].

### Increases in markers of tissue damage, remodeling and fibrosis

Our data indicate that there are increases in ethanolamine and glycerophosphoethanolamine in the PH lung (Fig 7), suggesting that elevated phospholipid degradation and membrane remodeling are occurring. Methylhistidines, produced by methylation of actin and myosin in muscle, provide an index of the rate of muscle protein breakdown. Thus, the increases in 1-methylhistidine, 3-methylhistidine, and acetyl derivatives (N-acetyl-3-methylhistidine) we observed (Fig 7) are suggestive of smooth muscle damage. We also observed increased 1- and 2-stearoylglycerophosphoglycerols (derived from the mitochondrial inner membrane component cardiolipin) and N-formylmethionine, a breakdown product of mitochondrially-encoded/synthesized proteins (Fig 7), which may reflect mitochondrial damage resulting from stress or the induction of cell death.

**Fig 8:**
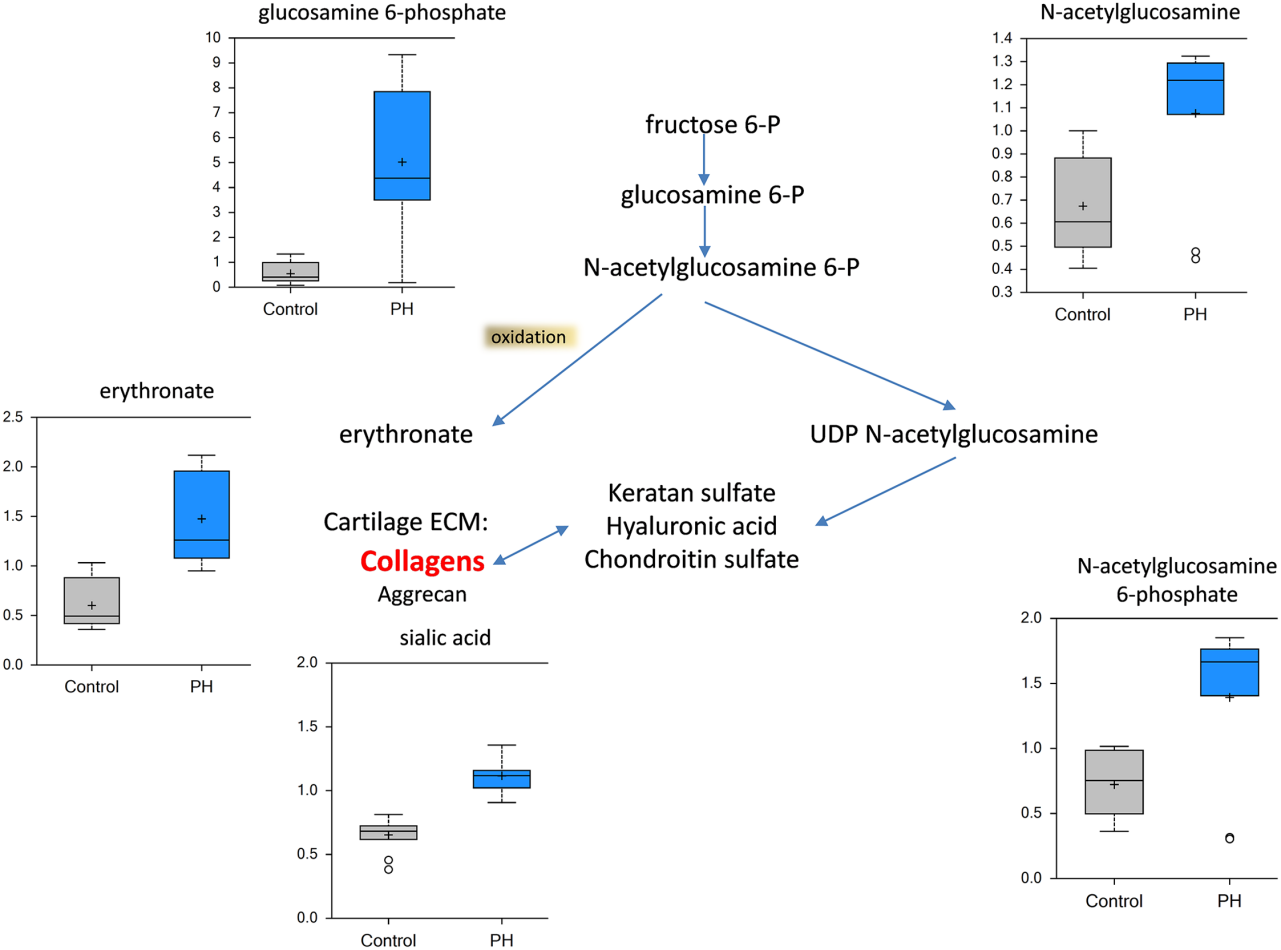
The extracellular matrix is remodeled in the MCT-treated rat lung. Initiating with fructose 6-phosphate, the glucosamine pathway contributes to extracellular matrix remodeling (center pathway). Glucosamine 6-phosphate, N-acetylglucosamine 6-phosphate, N-acetylglucosamine, sialic acid and the oxidation product erythronate were all significantly increased the pre-PH lung (N = 10, p<0.05).

**Fig 9:**
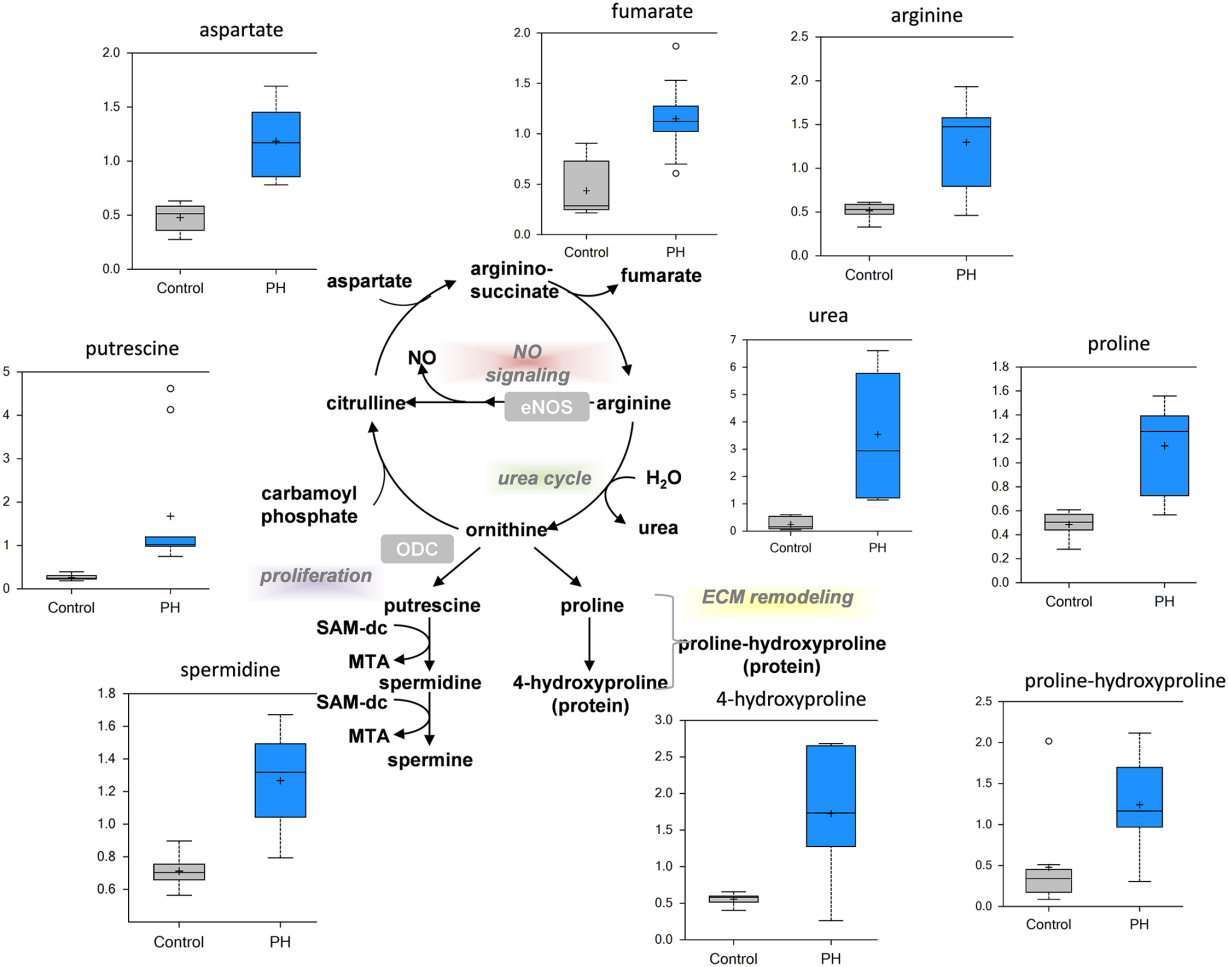
Disruption of arginine metabolism in the MCT-treated rat lung. The metabolic fate of arginine is complex being involved in NO signaling, the urea cycle, proliferation, and matrix remodeling (center pathway diagram). There is significantly increased urea production in the pre-PH lung indicative of increased arginase activity. Aspartate and fumarate, which are involved in arginine biosynthesis, are significantly increased in the pre-PH lung as is arginine itself. Polyamine metabolites are significantly increased in the pre-PH lung, indicative of increased cellular proliferation while the significant increase in proline pathway metabolites is suggestive of extracellular matrix remodeling (N = 10, p<0.05).

Following MCT-induced lung damage, a high molecular weight (HMW) hyaluronic acid (HA) polysaccharide component of the extracellular matrix is thought to undergo degradation to generate low molecular weight products. In support of this, we observed an increase in the HA metabolites glucosamine, N-acetylglucosamine 6-phosphate, and erythronate (generated by oxidation of N-acetylglucosamine) and in di- and tri-peptides (derived from protein degradation) in the pre-PH lung (Fig 8). These data are consistent with an increase in tissue remodeling occurring prior to the development of PH. We also observed that markers of collagen breakdown, trans-4 hydroxyproline and the collagen-derived peptide, Pro-OH-Pro and Ac-Ser-Asp-Lys-Pro-OH, a thymosin beta 4-derived anti-inflammatory and anti-fibrotic tetrapeptide [21, 22], were increased in the PH lung (Fig 9). Together these data are consistent with the concept that increased inflammation and fibrosis are occurring prior to the development of PH.

### Alterations in arginine metabolism

Nitric oxide (NO) synthases convert arginine to citrulline to generate NO. However, arginine can also be converted to citrulline (through ornithine) in the urea cycle via the activity of arginase. Although, complete urea cycle is available in liver, lung endothelium also has enzymes for arginine metabolism via urea cycle. Our data indicate that in the PH lung there is a significant increase in urea suggesting that L-arginine is mainly utilized by arginase (Fig 9). The arginase product, ornithine, required for polyamine and proline biosynthesis, is also increased in the PH lung (Fig 9). Ornithine is metabolized by ornithine decarboxylase (ODC) to generate polyamines, which are necessary to stabilize newly synthesized DNA and play a supportive role in in cell growth and division. We identified increases in the polyamine spermidine (Fig 9) suggestive that increases in the urea cycle may support cellular proliferation. Finally, elevated proline can be metabolized to hydroxyproline and its accumulation is thought to promote fibrosis [23]. Our data indicate that both 4-hydroxyproline and proline-hydroxyproline are increased in the pre-PH lung suggesting that a fibrotic pathway is being activated. Our recent data support this concept [24].

### Alterations in glutathione biosynthesis

**Fig 10:**
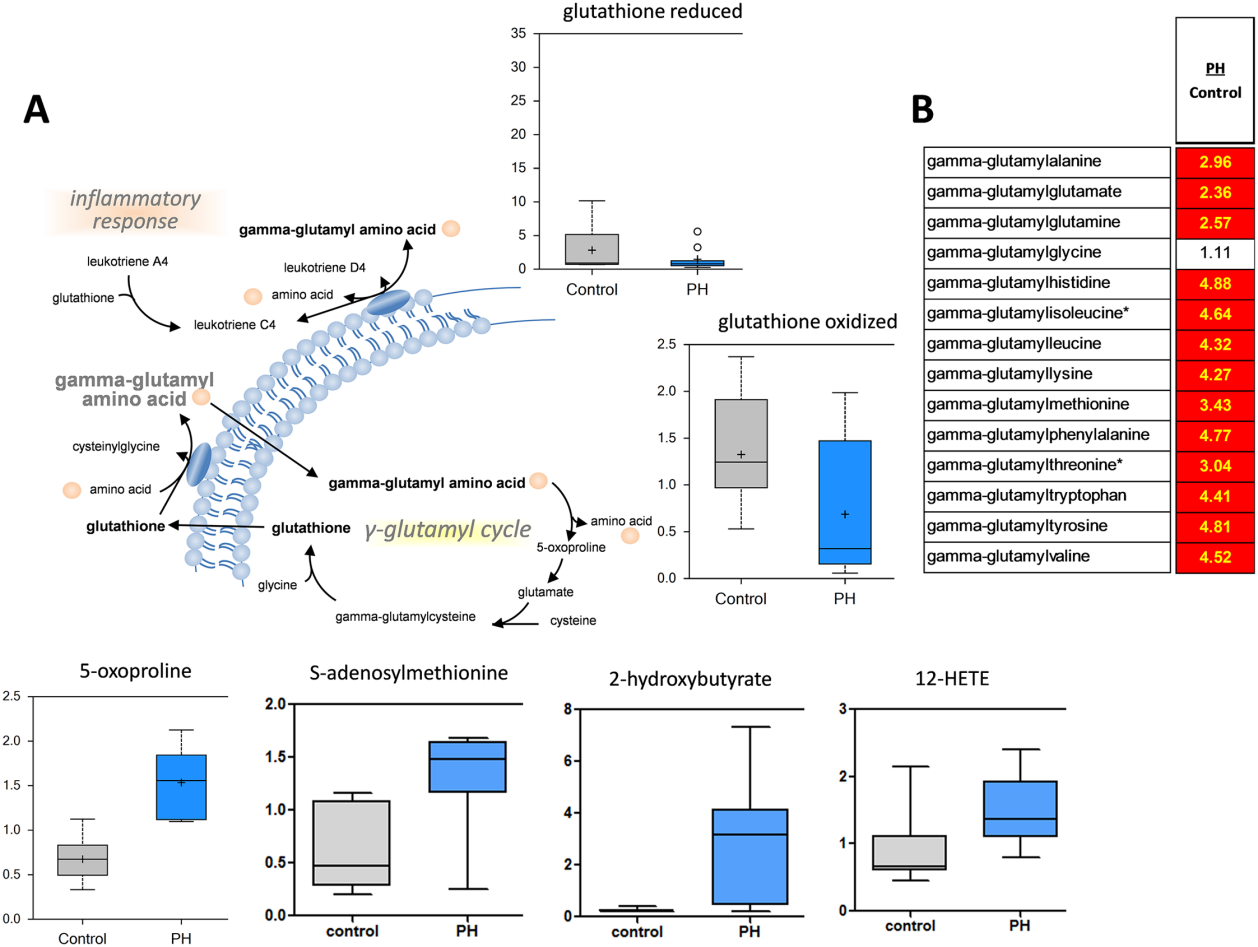
Evidence of a redox imbalance in the MCT-treated rat lung. Despite an increase in glutathione recycling, as indicated by increases in the 5-oxoproline metabolite, both reduced and oxidized glutathione are significantly decreased in the pre-PH (A), indicative of increased oxidative stress. The ratio between pre-PH and control metabolites demonstrate significant increases (red boxes) gamma-glutamyl amino acids (B). Activation of the gamma-glutamyl cycle is usually associated with an increased inflammatory response (N = 10, p<0.05).

We detected significant decreases in reduced glutathione (GSH) in the pre-PH lung that may reflect a disruption in redox homeostasis (Fig 10A); indeed, cystine (an oxidative product of cysteine), methionine sulfoxide and N-acetylmethionine sulfoxide (products of methionine oxidation) were all increased in pre-PH rats. Metabolites associated with the conversion of methionine to cysteine, such as S-adenosylmethionine (SAM), were also increased (Fig 10A), with decrease in S-adenosylhomocysteine suggestive of a high demand for glutathione. Moreover, SAM is involved in methylation of histamine by Histamine N-methyltransferase and our data (S1 Table) indicate high levels >600 fold of 1-methylhistamine accumulation in PH lungs. Increased levels of 2-hydroxybutyrate (AHB), which is produced as a byproduct when cystathionine is converted to cysteine in times of high glutathione demand (such as in response to an oxidative environment), were also identified in the pre-PH lung (Fig 10), while increases in ophthalmate and norophthalmate, a tripeptide analogue of GSH produced by glutathione synthase in which cysteine has been replaced by 2-aminobutyrate, are also consistent with increased demand for glutathione synthesis. Elevated levels of gamma-glutamyl amino acids (AA, Fig 10B) were accompanied by increases in 5-oxoproline (Fig 10A) and may reflect increased gamma-glutamyl AA exchange to replenish GSH. Finally, trends in increased tocopherols and carnosine (a histidine-derived dipeptide with anti-oxidative capacity), and significant increases in 12-HETE (which is generated by free radical oxidation of arachidonate, Fig 10) are consistent with increased oxidative stress.

## Discussion

**Fig 11:**
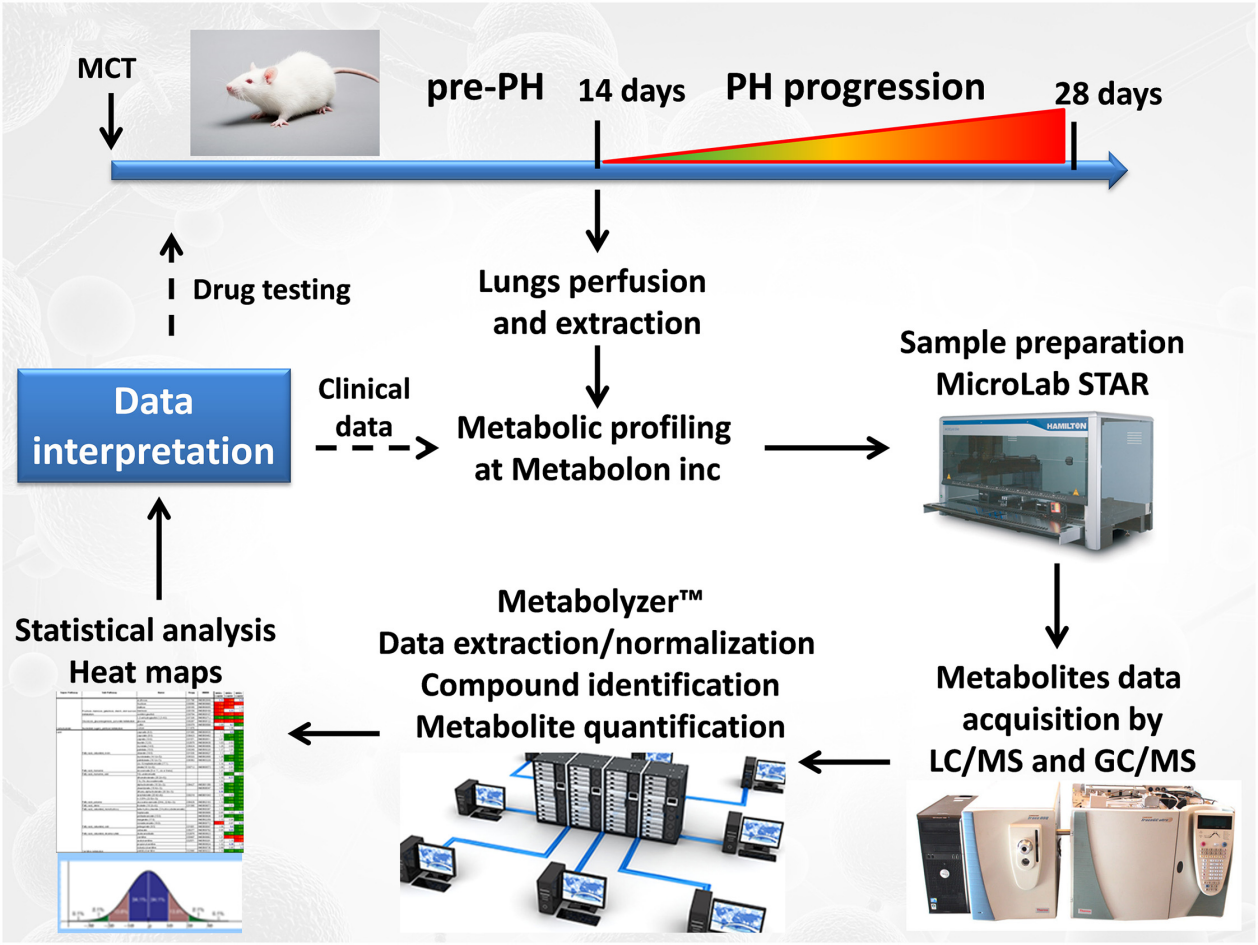
The metabolic study platform. Rats received MCT at day 0 and developed severe PH at day 28. However, lungs were collected for metabolomics at day 14 to monitor metabolic changes that precede pathological changes in the lung and heart. Lungs were perfused, collected and shipped to Metabolon inc for metabolic profiling. Sample preparation was done at Metabolon as described in the methods. Data from LC/GC MS were obtained and analyzed using the proprietary Metabolyzer™ software package. After statistical analysis and heat map generation, metabolic pathways altered 14 days after MCT injection were compared to control rats. In future (dashed arrows) this platform will be tested as a predictor for drug efficacy in the MCT and Sugen/hypoxia models of PH. Also, we will identify metabolite “fingerprints” that can be tested in clinical conditions for early PH diagnosis.

Pulmonary hypertension is a fatal disease characterized by pathologic vascular remodeling leading to right-sided heart failure. The monocrotaline (MCT)-induced rat model of PH has been used to dissect physiological and molecular aspects of this disease. Following injection, MCT is metabolically activated to a pyrrolizidine alkaloid in the liver; this compound exhibits extensive toxicity toward pulmonary endothelial cells, resulting in decreased barrier function, edema and eventually fibrosis. As a result, pulmonary vascular resistance increases and the right ventricle of the heart compensates by hypertrophy, leading to its eventual failure. Our hemodynamic data indicate mild pulmonary vasoconstriction with attendant changes in heart function/structure 14 days after MCT injection, whereas, 28 days after MCT injection the disease had progressed to a severe stage. Thus, to evaluate the metabolic changes at the early stage of PH, we conducted a global metabolic profiling study 14 days after MCT injection to identify biomarkers that changed prior to the development of PH. The metabolic platform is described in Fig 11. Our data indicate that 14 days after the injection of MCT, but prior to the development of PH [16] there is already a switch to glycolysis and a reduction in mitochondrial beta oxidation as reflected in the accumulation of glycolytic intermediates and products and reductions in acyl-carnitine long-chain fatty acid metabolites. This Warburg like effect, that underlies the proliferative phenotype of cancer cells, has been recently linked to the development of pulmonary hypertension [25]. In support of our data in the MCT-exposed rat, PH patient metabolomic data also observed a significant elevation of glucose and fructose 6-phosphate levels [7]. However, the changes in PH patients were less pronounced [7]. Underlying mechanisms of glycolytic switch in PH and possible treatments have been the focus of recent studies [26–29]. We have previously reported that carnitine shuttling of long-chain fatty acids is disrupted in a lamb model of PH [10]. This appears to occur secondary to increased endothelial NOS uncoupling, and subsequent high level of peroxynitrite generation in the lung results in carnitine acyltransferase nitration and inhibition [10]. Interestingly, this decrease in fatty acid beta-oxidation was also observed in humans with PH [7]. In this study we also found that several conjugated carnitine-fatty acid intermediates were decreased in the pre-PH lung suggesting that decreased beta-oxidation may be involved in the development of PH. As restoring carnitine homeostasis has been shown to resolve endothelial dysfunction [10, 18, 19] this may also be a potential therapeutic target in PH.

Another aspect of PH etiology is a contribution of inflammatory pathways in the development of the disease. It has been recently shown that fibroblast-macrophage interactions in PH lead to the activation of the inflammatory response through the secretion of cytokines and chemokines such us IL-1β, IL-6 and VEGF-A [30, 31]. Interestingly, the changes that occur in fibroblasts and macrophages during the development of PH are also associated with preferential aerobic glycolysis [31]. The pro-inflammatory damage associated molecular pattern (DAMPs) protein HMGB1 was also found to be a contributing factor in the development of PH through its ability to activate the TLR4 receptor [32]. Our data in MCT-treated rats are indicative of indoleamine 2,3-dioxygenase (IDO) activation as we observe significant accumulation of both kynurenine and kynurenate. This tryptophan degradation pathway can be activated by several inflammatory molecules such as TNFα, IL-6, and IFNγ [33, 34]. This may occur through a NFκ-B-mediated signaling pathway. This is consistent with other studies that attributed TLR4 receptor and fibroblast/macrophage activations in the pathogenesis of PH [31, 35]. Another spectrum of inflammatory pathway activation we observed in MCT rats involved the production of pro-inflammatory eicosanoids. The balance between omega 3 and 6 fatty acids is very important for inflammatory response and cytokine production in lungs [36]. Although, both are polyunsaturated fatty acids, omega 3 exhibits anti-inflammatory properties [37, 38] whereas omega 6 generally promotes inflammation [39, 40]. Our data in MCT lungs suggest there is disruption of the 6:3 ratio with an increase in total omega 6 pro-inflammatory fatty acids, specifically arachidonic acid. The formation of prostaglandins from arachidonic acid via cyclooxygenase results in an inflammatory microenvironment that attracts and stimulates immune cells [41–43]. Interestingly, treatment with a lipid emulsion containing omega-3 fatty acid in newborns with persistent PH increased left pulmonary blood flow by 30% and decreased pulmonary vascular resistance by 28% [44]. This suggests that restoring the balance between omega 6 and 3 fatty acids could be a potential new therapeutic target in PH.

PH is also associated with vascular and right ventricle fibrosis [45, 46]. Damage to the vascular wall results in remodeling that involves proliferation as a first step, which then progresses to fibrotic substitution in damaged tissues. This promotes increased stiffness of the lung vasculature [46]. The metabolic profile we observed in pre-PH lungs showed increased levels of glucosamine and its derivatives as well as hydroxyproline. These metabolites likely contribute to excessive extracellular matrix (ECM) remodeling. ECM remodeling is known to contribute to both fibril formations during fibrosis [47] and in the invasiveness of proliferating vascular wall cells into the parenchyma resulting in plexiform lesion formation [48]. Importantly, the glucosamine pathway starts from elevated levels of fructose-6-phosphate, which is upregulated in PH lungs due to a glycolytic switch in the cell’s metabolism. Thus, the glycolytic switch in PH can alter the proliferation of vascular cells, leading to the activation of inflammatory cells and ECM remodeling producing both fibrosis and plexiform lesions.

Endothelial NOS dysfunction is well characterized in PH [49–52]. Increased uncoupling of eNOS due to different factors—elevated levels of ADMA [53, 54], reduced association with Hsp90 [55], decreased tetrahydrobiopterin levels [56], oxidation of tetrathiolate cluster by hydrogen peroxide, nitric oxide or peroxynitrite [57–59]—reduce consumption of arginine through the NO pathway leading to vasoconstriction in pulmonary artery [54, 60]. The upregulation of arginase, which competes with eNOS for the same substrate—arginine, increases the consumption of arginine via the urea cycle [61, 62]. We identified a robust (~14-fold) upregulation of the urea pathway in pre-PH lungs. The downstream product of the urea cycle, ornithine is able to contribute to proliferation through polyamine biosynthesis and to fibrosis via the proline pathway. Both these pathways are increased in pre-PH lungs, suggesting that increases in enzymatic reactions downstream of ornithine may be preventing the normal conversion of ornithine back to citrulline and this could lead to the NO pathway being bypassed. However, it is also important to note that citrulline from the NOS reaction is situated in the caveolae micro-compartment, which possesses the enzymes (ASL and ASS) necessary to regenerate arginine at the NOS site. Whereas, citrulline produced by the urea cycle is inside the mitochondria, which would have to be translocated back to caveolae in order to feed into NOS cycle. It is unclear whether this occurs and is part of the arginine-paradox [63, 64] that suggests that there may be different arginine pools within the cell which are available only to NOS or arginase but not both.

In conclusion, results from this global metabolic profiling study revealed number of metabolic alterations in the pre-PH rat lung. Our data suggest a shift in energetics toward glycolytic processes and this may feed forward into the induction of inflammation, oxidative stress and fibrosis that are observed during the progression of PH. Interestingly, we observed a great deal of similarity between the metabolic changes in the MCT-model compared to previous metabolic profiling in humans with PH [7]. Thus, despite it failing to recapitulate the structural changes associated with advanced PH in humans, the MCT model may still serve as a useful pre-clinical model of PH. Our metabolic profiling of an early PH stage may lead to the identification of metabolite “fingerprints” that can be used to diagnose PH, monitor treatment efficacy, or be used as key metabolites to follow when testing new therapeutic regimens.

## Supporting Information

S1 Table Metabolite changes in MCT-treated rat lung. The table represents chemical name, ratio between PH and control group, p and q value for each metabolite in the study.
(XLSX)
